# Open biomedical pluralism: formalising knowledge about breast cancer phenotypes

**DOI:** 10.1186/2041-1480-3-S2-S3

**Published:** 2012-09-21

**Authors:** Aleksandra Sojic, Oliver Kutz

**Affiliations:** 1 European School of Molecular Medicine; European Institute of Oncology; University of Milan; Milan, Italy; 2 Research Center on Spatial Cognition (SFB/TR 8), University of Bremen, Germany

## Abstract

We demonstrate a heterogeneity of representation types for breast cancer phenotypes and stress that the characterisation of a tumour phenotype often includes parameters that go beyond the representation of a corresponding empirically observed tumour, thus reflecting significant functional features of the phenotypes as well as epistemic interests that drive the modes of representation. Accordingly, the represented features of cancer phenotypes function as epistemic vehicles aiding various classifications, explanations, and predictions. In order to clarify how the plurality of epistemic motivations can be integrated on a formal level, we give a distinction between six categories of *human agents as individuals and groups *focused around particular epistemic interests. We analyse the corresponding impact of these groups and individuals on representation types, mapping and reasoning scenarios. Respecting the plurality of representations, related formalisms, expressivities and aims, as they are found across diverse scientific communities, we argue for a pluralistic ontology integration. Moreover, we discuss and illustrate to what extent such a pluralistic integration is supported by the distributed ontology language DOL, a meta-language for heterogeneous ontology representation that is currently under standardisation as ISO WD 17347 within the OntoIOp (Ontology Integration and Interoperability) activity of ISO/TC 37/SC 3. We particularly illustrate how DOL supports representations of parthood on various levels of logical expressivity, mapping of terms, merging of ontologies, as well as non-monotonic extensions based on circumscription allowing a transparent formal modelling of the normal/abnormal distinction in phenotypes.

## Introduction

In this paper, we keep in line with two different, yet related traditions. We base our work closely on the position of perspectivism, as developed in the philosophy of science (for instance in [1,2]), whilst on the logical side we endorse Carnap's principle of 'logical tolerance' and the corresponding pragmatic version of logical pluralism [3-5] that ensues. Perspectivism accepts the position that human agents can never have a completely neutral and perspective-independent picture of 'the world', i.e. 'a view from nowhere' [1,2,6-9]. Rather, every scientific representation is defined by its research context and particular questions that scientists address [10,11]. Moreover, both traditions, perspectivism and logical pluralism, reconcile with a broader pluralistic stance [12-15] in philosophy of science and epistemology. However, we label our pluralistic stance as perspectivism because we believe it enables us to give a fruitful account of scientific practices, without committing to any particular metaphysical position. Therefore, following van Fraassen's view that

What scientific representation is and how it works is everyone's concern, and there we may find a large area where more general philosophical differences need make no difference. ([1], p. 3)

we focus our analysis on the level of perspective-driven scientific representations.

Indeed, this paper itself provides a view on phenotype representations from various perspectives. In the first part of the paper, we discuss the concept of phenotype through a number of examples, illustrating a variety of phenotype representations that co-exist in biomedical practice. From a position of perspectivism, we explain this heterogeneity of representations as originating in a huge variety of epistemic and pragmatic interests, distributed across and within biomedical sub-domains. Through the case of breast cancer research, we briefly clarify how various epistemic groups within the biomedical community, having different perspectives on the cancer related problems, produce domain specific representations. We emphasise a tight connection between 1) the epistemic and pragmatic interests of the agents who are building representations and 2) a plurality of the corresponding representations, thereby arguing that the heterogeneous representations reflect a plurality of domain interests. In order to resolve if and how these diverse interests and heterogeneous representations of biomedical and ontological communities can get integrated into a knowledge base, we distinguish epistemic agents as groups and individuals involved in ontology building. Furthermore, we specify similarities and differences among the groups regarding the representational means, the degree of formalisation, representational and semantic explicitness used in representation, as well as related knowledge base types. The remainder of the paper relates the representational pluralism, as an unavoidable part of the scientific practices, with a logical pluralism. We argue that not only the distributed epistemic and pragmatic interests influence various forms of scientific representation and reasoning, addressing the problems through diverse levels of granularity and expressivity, but the plurality of reasoning also converges into diverse kinds of logical formalisms, resulting in numerous 'most suitable languages' that may be used to model some domain formally. Therefore, we argue that the distributed ontology language (DOL) currently best fits the scientific practices composed of distributed epistemic and pragmatic interests. The paper concludes with a discussion of how DOL can support the interoperability of distributed ontologies, inter-connecting knowledge domains, while preserving domain specific needs and semantics. In particular, we illustrate this by sketching a modular, formal specification of mereology using ontology languages of increasing expressivity and by discussing how the various modules may be re-used in bio-medical ontologies with different purposes. Moreover, we re-use the mereology ontology in an OWL ontology extended with circumscription (which is a feature of DOL), modelling the normal/abnormal distinction in breast cancer related phenotypes.

## Perspectivism in phenotype representation

### Biomedical representations of phenotypes

In general terms, a phenotype is defined as a set of features of an organism that emerges as a result of interactions of its genetic material (specified as genotype) and the environment (see e.g. [16,17]). While the genotype of an organism is the class to which that organism belongs as determined by the description of the physical material made up of DNA passed to the organism by its parents, the phenotype of an organism is the class to which the organism belongs as determined by the description of the organism's physical and behavioural characteristics [17]. So, a person's genotype belongs to the class 'human' thanks to its inherited genetic material, description of which, despite of individual variations, fits to the general description of genome that is typical for the species 'homo sapiens'. The long lasting debate on species classification illustrates best that an agreement on what *the *typical features are, even on a gene level, is not a trivial task. We therefore consider *typical *features in terms of a conventional agreement at the current stage of scientific understanding. A person's phenotype includes features such as its size, its shape, its metabolic activities and its patterns of movement and behaviour. Some of these features can be selected to describe a *typical *human phenotype. In the case of anatomical descriptions, a canonical human anatomy represents a human phenotype as the organism composed of parts such as legs, arms, organs, cells etc. However, phenotypic features highly vary among individuals, and which particular features will be selected to classify individuals depends on classificatory aims. For example, a phenotype can be described through its structural, functional, or dispositional features, or through some quality such as eye colour [18].

### Phenotypes of disease

The representation of phenotypes plays an important role in clinical and biomedical knowledge, aiming at describing disease, assigning diagnoses and recommending therapies. Therefore, phenotypes are of particular interest to the biomedical expert if they include features that describe aberrances from what is considered a 'normal' phenotype. Moreover, exactly the aberrant features play a crucial role in the classification of a disease. A disease usually gets characterised through a distinction between 'normal' and 'abnormal' phenotypes, where 'abnormal' phenotypes serve as the marks of disease. The 'abnormal' phenotypes associated with a disease are labelled as *phenotypes of disease *(PD). However, the questions of what is 'abnormal' and *what *should be considered as a phenotype of a disease and *how *such a phenotype should be represented are rather contentious [18]. Clearly, the choice of how a PD should be represented is *normative *and *context dependent*. Consider the case of breast cancer and BRCA1 and BRCA2 gene mutations. In the age of genomic medicine, the very definition of disease has changed introducing a new kind of asymptomatic diagnosis. So, the carriers of BRCA mutations, without having developed any signs of breast cancer, still have a likelihood of 40-80% for developing an aggressive cancer phenotype during their life span [19]. The establishment of preventive treatments such as prophylactic surgery, chemoprevention and screening designed for the BRCA mutation carriers demonstrates that carriers of the mutated genes are indeed treated as patients [20]. Genomic medicine, thus, shifts the focus of PD from a traditional organ level approach to the gene level, treating apparently healthy people as 'patients'. For, the 'normal' breast phenotype in a BRCA mutations carrier will be irrelevant in the light of knowledge about 'abnormal', fine-grained phenotypes related to the gene expression patterns of the mutated genes. Since such a phenotype is classified as PD based on a disposition to develop the disease, the BRCA mutations carriers can get assigned a *dispositional *PD, according to the phenotype classification proposed in [18].

### Representing phenotypes in research contexts

Although these new directions in biomedicine aim at an integration of clinical and biological knowledge, the requirements across biomedical sub-domains significantly vary. So, a clinician will have different criteria for the representation of a phenotype than a molecular biologist. Regarding the goals of a discipline and the research context, information that is relevant for a clinician does not need to satisfy the needs of a molecular biologist who is mostly interested in phenotypic information about the molecular mechanisms associated with a disease. Likewise, features of a phenotype such as 'obesity', although clinically significant for breast cancer risk assessment, are excluded from the molecular description of a phenotype. Moreover, as we will illustrate by an example, the representations that a molecular biologist is typically interested in prioritise the explanatory role of the selected features, while the clinical representations usually aim towards clinical usability such as diagnosis assessment and therapy choice. As a result, heterogeneous representations and classifications of breast cancer phenotypes are employed in clinical and biomedical practice [21-23].

We will here not go into a detailed discussion of the philosophical debate on 'scientific representation'. For the purposes of this paper, in short, our approach is in line with Suárez's view which specifies two general, but necessary conditions that any scientific representation needs to satisfy. Namely,

*A *represents *B *only if (i) the representational force of *A *points towards *B*, and (ii)

*A *allows competent and informed agents to draw specific inferences regarding *B*. [[24], p. 773]

So, for instance, a mathematical, a pictorial, or a logical representation are the potential sources for an inference about a target. If *A *leads a scientist to an inference about *B *(a phenotype), then *A *will be a representation of *B*.

In light of this view of representation, concerning ways phenotypes can be captured by various technological and scientific tools, *representations of PDs *will include images acquired by technologies such as ultrasound, X-ray, and microscopy of histopathological samples. Moreover, representations of PDs are not limited to visual representations of a tumour, but include mathematical equations [25], statistical graphs, molecular markers, microarrays data [26], and the phenotype specific protein interactions [27], thus describing PDs according to the needs of and knowledge about a particular domain aspect. That is to say, a representation reflects which aspects of knowledge have been targeted by the representation and for what purpose. Differential equations of a mathematical model aim at representing cancer phenotypes whilst modelling, for example, carcinogenesis' dynamics. A PD is sometimes represented by the equations that model the response to a particular treatment, thereby playing a *predictive *role [25]. Some other representations have primarily a *heuristic *role, using mechanistic and causal models to represent various aspects of a PD, which can support the *understanding *of carcinogenesis or a specific response to a therapy [28]. Accordingly, a representation reflects a scientist's choice to model a certain subset of the domain knowledge for a particular purpose. Not every PD representation can equally satisfy all possible explanatory, predictive, and pragmatic needs, even within a single domain. Rather, various types of representations have been employed to model diverse aspects of a domain problem. Therefore, 'choosing a representation' might be considered a highly intentional act [8].

However, a representation such as a histopathological image will not, itself, represent any knowledge unless it gets interpreted. Knowledge within a domain is *explicitly *represented only if the representations get systematically connected with related interpretations, knowledge claims, and reasoning over the representations. As a representation may have various interpretations, expressing diverse aspects of knowledge about what is being represented, it can also get assigned diverse knowledge claims and mapping relations. Therefore, besides heterogeneity of PD representations, biomedical ontologies have to deal with a heterogeneity of reasoning about PDs, comprising different kinds of formal (or logical) representations as well as various types of reasoning. Conversely, the intended reasoning methods or types over PDs also influence the choice of representation of PDs because such representations are mediated by domain specific methods and interventions, employed in the imaging, measuring of the gene expression and other diagnostic and experimental techniques [29]. Consider, for example, clinical representations of breast cancer that go beyond the tumour imaging representation. According to the standards of the TNM classificatory system [21], the clinical classification of tumours considers tumour size (T), lymph nodes involvement (N), and presence of metastasis (M). Of course, tumour size is just one feature and is not sufficient for the characterisation of the tumour type. Cancer is a dynamic and complex disease of an organism and the PD representations, therefore, go beyond the characterisation of a tumour captured in a static picture. For example, knowledge about lymph nodes' status or proliferation marker KI-67 provides additional information about a tumour's phenotype. Likewise, tumour markers provide a view on the PDs through the specific interventions on the representation. A detection system can target a gene or a protein of interest, staining the samples in order to produce a clinically useful PD representation. Had the estrogen receptor (ER) been detected, the PD would have been described as an ER+ positive tumour, which significantly differs from an ER- (negative) tumour, which does not respond to the endocrine therapy [30]. Thus, the therapeutic criteria often play a crucial role in the specification of the tumour phenotypes.

### Phenotype ontologies

Phenotype ontologies aim at capturing phenotypes formally. Developing phenotype ontologies has been recognised as a particularly challenging task for ontologists because of the complexity and heterogeneity of features used to describe a phenotype. Several approaches have been proposed to describe phenotypes formally, some of which use 'qualities' as attributes assigned to 'entities' [16,31,32]. In particular, phenotype ontologies encounter the problem of having to represent features that are not 'normal' in terms of canonical anatomy. For instance, Hoehndorf et al. [18] have introduced two disjoint predicates, C (canonical) and NC (non-canonical) in order to support 'abnormal' phenotype representations. This approach is part of a general framework that categorises the concept of phenotype as 'Phene' into the classes of 'object phene' and 'process phene', while 'object phene' is subdivided into the classes 'structural phene', 'qualitative phene', 'dispositional phene' and 'participatory phene', including further subdivisions [18]. The phenotypic relations of the Phene ontology are formalised using the OWLDEF method [33] in order to endow phenotype representations with explicit semantics and to enable interoperability with existing domain ontologies. An advantage of such an approach compared to the entity-quality (EQ) characterisation of phenotypes [16] is that it can support inference among the pheno classes that are imported from domain ontologies such as FMA, while EQ has limited interoperability and inferential potentials as it considers all phenotypic features as qualities (see [18]). The introduced Phene relations such as CC-pheneOf-lacks-part nicely describe phenotypes that are 'abnormal' due to absence of anatomical parts. In contrast to this, we address the case where the quantity of a part (e.g. the HER2 protein which is present in the cell membrane in both 'normal' and 'abnormal' phenotypes) makes a qualitative difference, resulting in an 'abnormal' phenotype. We here deal with this problem using a non-monotonic mereology specification.

#### Merging perspectives of distributed ontologies

Regarding biomedical ontologies in general, there have been particular efforts in creating alignments among the internationally recognised biomedical vocabularies [34-36], e.g. as represented in the form of a thesaurus (e.g. NCI, SNOMED), metathesaurus (UMLS), and biomedical classifications such as the International Classification of Disease (ICD), (Respectively, http://ncit.nci.nih.gov/, http://www.ihtsdo.org/snomed-ct/, http://www.nlm.nih.gov/research/umls/, http://www.who.int/classifications/icd/en/) with OBO foundry ontologies and other formal ontologies such as General Formal Ontology GFO [37], GALEN [38], and the Dolce ontology [39]. The OBO foundry includes various ontologies each of which covers some aspects that are relevant for the representation of phenotypes. For instance, the Human Disease Ontology (DO) (http://www.obofoundry.org/cgi-bin/detail.cgi?id=disease_ontology) aims to represent disease related concepts. For the representation of breast cancer phenotypes some of the key terms from DO include 'cancer', 'breast cancer', 'Her2-receptor positive breast cancer', 'female breast cancer', 'male breast cancer', 'estrogen-receptor positive breast cancer', 'estrogen-receptor negative breast cancer', 'progesterone-receptor positive breast cancer', 'progesterone-receptor negative breast cancer', and 'triple-receptor negative breast cancer' (The DO terms' identifiers listed are, respectively, DOID:162, DOID:1612, DOID:0060079, DOID:0050671, DOID:1614, DOID:0060075, DOID:0060076, DOID:0060077, DOID:0060079, DOID:0060081). The represented concepts in DO are organised in a hierarchical structure using the is_a relation. However, the hierarchy of DO concepts is not sufficient to represent breast cancer phenotypes. Although 'cancer' and 'breast cancer' within DO are specified by their lexical definitions, DO does not represent explicitly the defined concepts. That is to say, definitions of cancer as 'disease of cellular proliferation that is malignant and primary, characterized by uncontrolled cellular proliferation, local cell invasion and metastasis' and of breast cancer as 'a thoracic cancer that originates in the mammary gland' do not represent how, for example, 'cancer', 'breast cancer', and 'Her2-receptor positive breast cancer' relate to each other in a more specific way than what the relation of subsumption can communicate. A more specific representation of a breast cancer phenotype asks for concepts and relations that DO does not contain. As it has already been recognised in the case of prostate cancer [40] and phenotype ontologies in general [18], abreast cancer phenotype ontology needs to combine various other ontologies each of which is designed for a specific purpose. The Foundational Model of Anatomy (FMA) aims to represent anatomical concepts and relations in a canonical way. Thereby, FMA does not include 'abnormal' anatomical features. However, FMA can provide a phenotypic characterisation of a canonical breast anatomy and its lymphatic system that is lacking in DO. Likewise, the Phenotypic Quality Ontology (PATO) (see http://www.bioontology.org/wiki/index.php/PATO:Main_Page) provides representations of the qualities that are lacking in FMA and DO. Even if DO includes some of the quality terms such as 'aggressive', the particular qualities are not represented as separate concepts, rather they are just included in a definition or they make an inseparable part of a more complex concept such as 'aggressive periodontitis'. Particular qualities from PATO (e.g. 'abnormal', 'increased concentration', 'poorly differentiated', 'aggressive', 'progressive') (Respectively, PATO:0000460, PATO:0001162, PATO:0002106, PATO:0000871, PATO:0001818) can be easier reused in various contexts, facilitating automated inference. The Units of Measurement Ontology (UO), on the other hand, provides concepts such as 'count', 'molecule count', 'percent', 'length unit' (Respectively, UO:0000070, UO:0000192, UO:0000187, UO:0000001), which are important for molecular and clinical descriptions of breast cancer phenotypes. In a similar manner, an ontology that aims to represent breast cancer phenotypes needs to consider, *inter alia*, the concepts 'assay' and 'specimen' (Respectively, OBI:0000070, OBI:0100051) from the Ontology for Biomedical Investigation (OBI) (see http://obi-ontology.org/), 'HER2 signaling pathway' and 'regulation of Neu/ErbB-2 receptor activity' (Respectively, GO:0038128, GO:0060726) from the Gene Ontology (GO), 'Tyrosine kinase-type cell surface receptor HER2' (CCO:B0005540) from the Cell Cycle Ontology (CCO) or from the Protein Knowledgebase (UniProtKB:P04626), and 'tyrosine kinase inhibitor' (CHEBI:38637) from Chemical Entities of Biological Interest (ChEBI [41]). Concerning the clinical management of patients, the information from a drug bank should also be considered in the description of a phenotype, allowing a specification of how, for instance, HER2 positive breast cancer behaves in response to the 'Herceptin' (DB00072) treatment. (E.g. http://www.drugbank.ca/drugs/DB00072; Herceptin in the Drug Bank database has not yet been organised in an ontology, but the references to the related ontologies are provided.)

The presented variety of ontologies properly illustrates the very definition of **ontology **as a specification of a conceptualisation that represents a selected aspect of the world for a particular purpose, as given by Gruber [42]. Accordingly,

[. . .]When the knowledge of a domain is represented in a declarative formalism, the set of objects that can be represented is called the universe of discourse. This set of objects, and the describable relationships among them, are reflected in the representational vocabulary with which a knowledge-based program represents knowledge. [. . .] In such an ontology, definitions associate the names of entities in the universe of discourse (e.g., classes, relations, functions, or other objects) with human-readable text describing what the names mean, and formal axioms that constrain the interpretation and well-formed use of these terms. ([42], p. 908-909)

Each of the above mentioned ontologies (e.g. FMA, PATO, GO, ChEBI etc.) is designed for a particular purpose. Therefore, the represented concepts are selected and related among each other in a way that best fits the aims of the particular ontology. While some of the concepts from diverse ontologies can be mutually aligned, in other cases it is likely that the concepts will stay represented only in one domain (universe of discourse) and not in the others. An introduction of new concepts (e.g. introduction of GO and OBI concepts to FMA) would unnecessarily increase complexity and most likely it would result in numerous redundancies and inconsistencies. Therefore, a combination of various modules from domain ontologies, as proposed in e.g. [5,18,43], seems as the most viable solution when a particular task, such as the representation of breast cancer phenotypes has to be achieved. In our paper, we tackle just a segment of the problem as to how certain modules of FMA can be used in the representation of a breast cancer phenotype. Before we propose our model, we analyse how the diversity of domain interests influences the ontology design. The analysis of the epistemic groups involved in the ontology building emphasises a plurality of epistemic needs among the groups that our proposal attempts to deal with.

## Representing the plurality of domain interests

In the previous section, we presented a few examples of how sub-domain interests influence the representation of breast cancer phenotypes. Moreover, we demonstrated that specific perspectives on how phenotypes should be represented results in a proliferation of representations in biomedical practice. To reconcile how this variety of representations can be interoperable, we hold that instead of favouring a particular one, those different representations actually can be embraced as an array of perspectives, each of which may produce useful insights into the problem, and which thus have a systematic connection at a higher level of abstraction. In order to pursue our analysis of interoperability across sub-domain representations, in this section we analyse the epistemic groups that are using various representational means, while being involved in knowledge base building. A better understanding of the epistemic agents who are building representations will give an insight into how collaborative efforts evolve from implicit and less formal to explicit formal representations of knowledge.

### The collaborative interests

The organisation of huge amounts of heterogeneous data produced and analysed by various methodologies and diverse types of reasoning necessitates collaboration across scientific domains. An emerging issue of knowledge integration concerns the two apparently counteracting forces: *disciplinary division *that drives specialisation of knowledge on one hand, and *inter-disciplinary collaboration *on the other. Disciplinary specialisation allows a detailed analysis of specific problems within a research group, focusing on, for example, a particular protein that modifies gene expression, eventually resulting in the development of cancer. However, when knowledge acquired within a research group has to be integrated into the big picture of complex carcinogenic events, collaboration beyond the particular research domain is unavoidable. Collaboration among clinicians, molecular biologist, and bioinformaticians will show to be crucial for the application of molecular knowledge in clinics. Moreover, computer scientists and ontology engineers play an important role in providing the formal and technical means for knowledge integration.

A big promise of the integrative endeavour in contemporary science is that dispersed pieces of knowledge produced within one domain and not yet related to knowledge from some other domain, when being connected, can actually drive new inferences, produce new knowledge and a better understanding of a disease [44]. This old idea that knowledge can be expanded by connecting dispersed pieces of information gained particular importance with the development of information systems such as databases, semantic web technologies, and ontology tools which are mapping and representing concepts and sets of data across fields. Such information technologies allow data storage, a potential interconnection, and manipulation with a huge amount of heterogeneous types of data which goes beyond cognitive abilities of any particular expert. As knowledge organised in databases and ontologies represents the concepts in a computer readable form, the use of implemented reasoners enables an automated inference about represented information, e.g. for DLs FaCT++, Pellet, RacerPro, HermiT, etc. (see http://www.cs.man.ac.uk/~sattler/reasoners.html) and first-order and other logics see e.g. http://www.informatik.uni-bremen.de/agbkb/forschung/formal_methods/CoFI/hets/index_e.htm. So, in the case of cancer management, an integration of a patient's clinical data with information about the PD, patient's molecular profile, family history, presence of other diseases, life style habits etc. can help clinicians to assess the most appropriate, patient specific treatment. Likewise, when connected, various PD representations can enhance the understanding of the disease and improve clinical utility of the acquired knowledge.

### The epistemic groups

Information technologies and formal tools such as ontologies for knowledge representation (KR) aim at formally representing heterogeneous knowledge domains and different types of domain specific representations [5,43]. Concurrently, clinicians and molecular oncologists are trying to organise and apply the overwhelming and diverse knowledge about cancer biology. Can these interests of different disciplines meet in a constructive union, while preserving the domain specific representations and reasoning capabilities?

In this section, we outline some of the requirements for achieving such a level of interoperability. We begin by giving a comparative analysis of the distribution and character of knowledge involved in the integration of heterogeneous types of knowledge represented in knowledge bases (KBs). In particular, we distinguish *where*, *how*, and *by whom *knowledge is represented by characterising six epistemic groups, and by discussing how membership to a group impacts the representation as well as knowledge base types. Note that these groups exhibit rich interdependencies and partially overlap.

1. The characterisation of the epistemic groups starts with the societal demands for problem solving, such as, for example, the need for personalised breast cancer therapy [45,46]. The demands may be represented in the form of standards, platforms and funding policies [46,47]. In a democratic society, knowledge on this level can be represented as common or shared knowledge available to the members of society; knowledge can be distributed through various channels or common-sense KBs.

2. The second epistemic group to be discussed is at the level of an individual scientist whose 'knowledge base' is a collection of relevant background knowledge, here to be understood as cognitive representations placed in the mind, arguably, in the form of conceptual maps (see [48]). As far as the conceptualisation of a problem has not been communicated in an inter-subjectively accessible way, e.g. by means of language, the related representations stay private to the mind of a particular epistemic agent. Thereby, the representation of a cognitive conceptualisation is *implicit *[49]. Likewise, whilst an individual may assign a referent (an object, a term) to such an implicit representation, the semantics of the relation between the referent and the related cognitive content stays implicit, i.e. accessible only to the individual mind.

3. As the third epistemic group, we specify the scientific communities, each of which is composed of the specific disciplinary domain scientists (clinicians, molecular biologists, bioinformaticians etc.). This epistemic group establishes knowledge within a scientific community as a received view, having the form of *explicit *and *inter-subjective *representations expressed in the respective scientific languages, circulated through publications. Like in group (1), knowledge can be distributed in various ways. Contrary to the implicit conceptualisations (group 2), a community establishes shared conceptualisations that are made explicit by means of language.

A shared conceptualisation within a community, thanks to the shared terminology and the context of its use, enables distinctions between domain specific terms and their relations as an agreed upon meaning to represent domain knowledge. However, these shared representations are still local and specific to particular communities. Like in (2), the semantics of the terms and the mapping relations among them stay implicit. Namely, it is left to the interpreters of language to assign meaning to terms by a cognitive process which assigns referents and relations to the represented terms.

4. The fourth group comprises scientific communities formed around a particular problem (e.g. breast cancer). As the group contains multidisciplinary teams focused on a particular problem, knowledge will need to be coordinated in such a way that the used scientific terms and reference classes will conform with knowledge within diverse domains. For instance, the biomedical terms might be structured into networks of terms that represent how these terms are interrelated in the domain knowledge. Thus, collaboration here results in merging knowledge from different domains. The representation of the merged knowledge coming from different perspectives on the same problem might be a 'unified semantic map' (see group (2)) that serves as a semi-formal conceptual model and an intermediate step towards the KB and the formal ontology to be employed in KR (see e.g. [50]). Note that, as presented above, group (4) is heterogeneous in itself because it is composed of experts from various scientific fields (group 3), thereby producing a variety of breast cancer related representations. We will consider closer the domain heterogeneity in the following sections.

5. The fifth is the communities of logicians and ontologists who are formalising ontologies according to the needs and specificities of a particular field. Domain knowledge and the merged domain knowledge will be expressed as ontologies written in various formal languages (e.g. refining foundational ontologies such as DOLCE [39], BFO (see http://www.ifomis.org/bfo/), or GFO (see http://www.onto-med.de/ontologies/gfo/) etc. formalised in OBO, OWL, or first-order logic, etc.) As a shared conceptualisation gets enriched with formal specifications that have well-defined formal semantics [51], the semantics of the represented domain is *explicit*. Accordingly, related KBs will contain explicitly represented knowledge.

6. The sixth group involves computer scientists, programmers and engineers, who are designing databases and applying formal ontologies as well as various reasoning tools to large data sets. Technically, a representation built on top of a database involves types and mapping relations *structuring *the data, and can be considered as meta-data. Here, the representation integrates the types and mappings with instances (data). Epistemic accuracy of the mappings depends on how well the mappings correspond to the scientific knowledge and the empirical findings of the represented domain (e.g. breast cancer). In contrast to groups (2) and (3), knowledge in a KB is not scattered over various representational spaces or layers, but integrated into one. As the ontology mapping terms to instances provides the representational reference within a KB, both semantics and representation are explicit.

In Table 1, we show the epistemic agents as they are organised into the 6 groups, and illustrate to what extent they are involved in ontology and KB building. The hierarchical grouping of the agents is just an approximation, according to the representational means and the level of explicitness employed to represent knowledge. Of course, a real time process of knowledge organisation and distribution is much more complex than what this table can summarise. However, the table sketches some basic distinctions distributed across the specific representational domains. The epistemic groups are ordered according to the degree to which knowledge representations are made explicit, ranging from implicit and less formal to the most explicit formal representation of knowledge. Collaboration among the domain experts is presented as crucial for knowledge integration. A group of experts with a common interest is collaborating in establishing a shared conceptualisation [52] (group 4), which gets formalised (groups 5 and 6) according to the established standards that help them label and describe the domain of interest in an interoperable way [53]. Knowledge levels, groups, or layers have of course been discussed previously in the AI literature. For instance, Newell introduced an agent-based distinction between the 'knowledge level' and the 'symbol level' in [54], and [49,55,56] analysed layers in formal ontology design. In more detail, Brachman, in 1979, introduced a classification of the primitives used in KR systems at the time [55], distinguishing the following four levels: (i) 'Implementational', (ii) 'Logical', (iii) 'Conceptual', and (iv) 'Linguistic'. Guarino [49,56] added to these four layers yet another layer, namely the 'Epistemological Layer' for the primitives, situated between the 'Logical' and the 'Conceptual' layers. Our approach differs in that it mainly aims at distinguishing *human agents as individuals and groups *focused around particular epistemic interests, whilst analysing the corresponding impact on representation types. A more detailed analysis of the relationship to previous 'layering approaches' is left for future work.

**Table 1:** Organising knowledge: the epistemic agents and representational means

	Epistemic group	Representation type	Knowledge (base) type
I	Society	**Demands** Problem (e.g. patient, disease) Solution (e.g. diagnosis, prognosis, therapy) standards and funding policies	Common knowledge

II	Individual Scientists	**Cognitive conceptualisation** Implicit representation in mind Implicit semantics	Background knowledge of an individual scientist

III	Communities (clinical, biomedical, bioinformatical etc.)	**Biomedical claims** expressed in the scientific language - publications Explicit representation of domain knowledge Implicit semantics	Background knowledge of a scientific community
		**Terms as units of biomedical claims** Explicit representation of the terms - definition Implicit semantics	Distributed domain knowledge Various networks of biomedical terms

IV	Community (breast cancer)	**Model for an ontology** Explicit representation of a unifying conceptual model expressed in the scientific terms as a shared conceptualisation Semi-explicit semantics	Sub-domain knowledge problem related (**merging domains**)

V	Computer scientists Logics	**Ontology** Explicit formal representation of shared conceptualisation expressed in a formal language - formal ontology Explicit semantics	Formalised knowledge

VI	Computer scientists Engineering	**Mapping **ontology onto data records (metadata) Merged ontology model and information model - applied ontology Explicit representation and semantics	AI **Knowledge Base** **(KB)**
			
		**Data **(Instances) structured within database architecture Data models	

#### Ontology engineering and epistemic groups

Regarding the perspectivism in ontology representation, the distinction of the epistemic groups captures some basic similarities and differences among agents who aim at conceptualising and representing a problem. Namely, consider the following snippets from Gruber's definition [42, p. 908-909] of ontology (given above) as:

• 'simplified view of the world that we wish to represent for some purpose': an ontology as a technical artefact is not intended to cover the world in its entirety, but only chosen aspects of the world, on specific levels of abstraction, and for given purposes--largely independent of particular metaphysical positions such as realism and antirealism; here, group (4) will typically informally specify the relevant domain knowledge (e.g. a conceptual map of breast cancer phenotypes that integrates structural and functional features of a protein involved in carcinogenesis), whilst group (5) is in charge of establishing an agreement on how to formally codify this knowledge.

• 'committed to some conceptualisation': ontologies presuppose various decisions concerning ontological commitments. These originate partly in common sense knowledge (group (1)), precisifications given by members of group (2), and agreements as they are established in groups (3) and (4). Finally, the formal implementation of the ontological commitments is again left for groups (5) and (6), merging collaborative interests of (1)-(6).

• ' "what exists" is that which can be represented': ontological commitments are dependent on the expressive capabilities of selected representational formalisms. The choice of an adequate formal language can only be established as an interplay between logician (group (5)), computer scientist (group (6)), and the domain experts of (3) and (4).

• 'representational vocabulary' and 'human-readable text': there is a 'tension' between the logical vocabulary used, and the natural language concepts and terms it is meant to capture, and, in the case of e.g. breast cancer, various forms of scientific representations such as graphs, mathematical equations, images, 3D models etc. Reconciling this tension requires deep interaction between the various groups of domain experts and formal logicians and computer scientists.

• 'an ontology is the statement of a logical theory': on a technical level, an ontology is seen as equivalent to a logical theory, written in a certain formalism. Clearly, this task is for group (5), respecting the requirements of group (6).

The above remarks apply to a variety of formal languages used in ontology engineering. Indeed, heterogeneity of formal languages is particularly important in the life sciences, where size of ontologies and needed expressivity vary dramatically. For example, whereas weak (i.e. sub-Boolean) DLs suffice for the NCI thesaurus (containing about 45.000 concepts) which is intended to become the reference terminology for cancer research [57], other medical ontologies such as GALEN (see http://www.opengalen.org/) require the full expressivity of the OWL language (a decidable fragment of first-order logic), while foundational ontologies typically require at least full first-order logic (see [43]). Alternatively, new and more expressive languages to model complex biological processes have also been proposed [58].

An example of a heterogeneous combination of formalisms is discussed in [59], where it is shown that in order to adequately represent the spatial structure of molecules as they are described in chemical ontologies such as ChEBI [41], ontology languages need to be combined with formalisms such as monadic second-order logic.

We next illustrate how such diversity and heterogeneity is reflected in and how it originates from different group interests in the case of breast cancer phenotypes representations.

### A plurality of mappings and reasoning: the case of 'HER2'

In biomedical ontologies, metadata in the form of tags, annotation, or more generally documentation, is of particular importance. Indeed, many biomedical ontologies have an extremely shallow logical structure, namely consist only of taxonomies, or even just of sets of concepts, however accompanied with a rich set of metadata. It is clear that the separation of the epistemic groups introduced above has a direct impact on the kinds of annotations and metadata that can be expected to be generated. For instance, the particular scientific communities (groups (2) and (3)) need not associate identical sets of concepts as related to a term in use. When the Human Epidermal growth factor Receptor 2 (HER2, also known as ErbB2) is used as a tumour marker in the community of clinical oncologists, 'HER2' is related to the diagnostic terms. E.g. over-expression of *HER2 supports diagnosis *of *HER2 breast cancer*, which is described as an *aggressive tumour *with a *poor clinical outcome *and a *low likelihood of a long term survival*. On the other hand, among the group of molecular biologists 'HER2' is mostly used to characterise molecular processes such as the HER2 related *protein-protein interactions *that trigger the *carcinogenic events*. Of course, 'HER2' can serve as a link between the two domains. However, as interests diverge among and within disciplines concerning ways of describing a phenotype, distinguishing similarities and difference makers will vary among knowledge domains. So, HER2 will not be the same difference maker for a clinician and for a biologist. The main difference that will be relevant for a clinician will be a difference in the patients survival associated with the expression of HER2 [60]. The biologist who focuses on the cellular signalling pathways looks for, for example, a differential expression of the ErbB2 gene while comparing the phenotypes of two types of cell lines [61]. Consequentially, a justification of asserted similarities and generalisations asks for a different kind of evidence in diverse domains. Clinical evidence is acquired through survival analysis and clinical trials while biologists provide evidence through diverse experimental and explanatory methodologies [62]. Accordingly, the reasoning of the groups (2)-(4) influences the related mappings and justifications implemented by groups (5) and (6).

**Figure 1:**
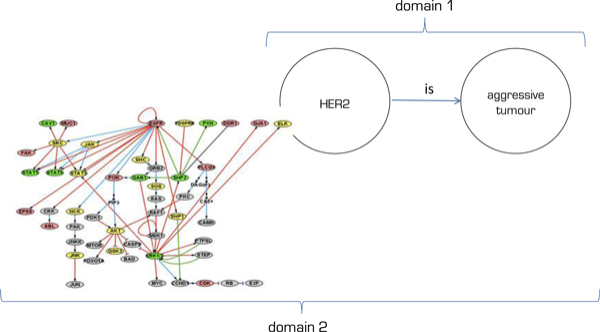
**Knowledge granularity**.

A relation between a term and its reference class gets its justification within domain knowledge as an adequate mapping relationship. The justification is expressed through claims that support the mapping relations. Regarding the previous example, 'HER2' will be mapped onto a 'bad prognosis' within clinical knowledge (see Figure 1, Domain 1), and the mapping will be justified by the statistical data retrieved from survival analyses. Likewise, biological knowledge provides an alternative mapping relation and a related justification for a mapping between 'HER2' and 'tumour aggressiveness', e.g. protein interaction pathways that result in cell proliferation and tumour aggressiveness (see Figure 1, Domain 2), captured, for instance, in GO, UniProt, and KEGG (see http://www.genome.jp/kegg/).

A detailed analysis of the mappings within and between knowledge domains asks for a multidisciplinary approach involving a community-based process of knowledge production [63,64]. A group of experts with a common interest is collaborating in establishing a shared conceptualisation [52] (groups 4 and 5), which gets formalised (group 5) and implemented (group 6) according to the established standards that help them label and describe the domain of interest in an interoperable way [53,65].

Below we will consider the example of HER2-positive phenotypes from a mereological perspective and also discuss the issue of normal vs. abnormal phenotype from a formal modelling perspective.

## A plurality of knowledge representations

Having presented and discussed the plurality of biomedical reasoning and related representations, this section focuses on plurality as it pertains to formal knowledge representation. A variety of languages is used for formalising ontologies. Some of these, such as RDF (mostly used for data), OBO and certain UML class diagrams (namely those avoiding qualified associations (amounting to identification constraints), *n*-ary relations (for *n *> 2) and stereotyping), can be seen more or less as fragments and notational variants of OWL (or first-order logic), while others, like F-logic and Common Logic (CL), clearly go beyond the expressiveness of OWL. To address the modelling problems that result from using concurrently this variety of languages, [66,67] lay the foundation for a distributed ontology language DOL, which is intended to allow users to use their own preferred ontology formalism whilst becoming interoperable with other formalisms, which we sketch next.

### The distributed ontology language DOL

OWL is a popular language for ontologies. Yet, the restriction to a decidable description logic often hinders ontology designers from expressing knowledge that cannot (or can only in quite complicated ways) be expressed in a description logic. A practice to deal with this problem is to intersperse OWL ontologies with first-order axioms, e.g. in the case of biomedical ontologies where mereological relations such as parthood are of great importance, though only partly definable in OWL. (Note that in the following, we adopt the completely formal position that an ontology is nothing but a formal theory in a given ontology language, and that an ontology language is any logical language that is considered suitable for ontology design by some community.)

However, these remain informal annotations to inform the human designer, rather than first-class citizens of the ontology with formal semantics and impact on reasoning. One goal of the DOL language is to equip such heterogeneous ontologies with a precise semantics and proof theory.

DOL faces the logical diversity not by proposing yet another ontology language that would subsume all the others, but by accepting this pluralism in ontology languages and by formulating means (on a sound and formal semantic basis) to compare and integrate ontologies that are written in different formalisms. This view is a bit different from that of unifying languages such as OWL and Common Logic (CL), which are meant to be "universal" formalisms (for a certain domain/application field), into which everything else can be mapped and represented. While such "universal" formalisms are clearly important and helpful for reducing the diversity of formalisms, it is still a matter of fact that no single formalism will be the Esperanto that is used by everybody. It is therefore important to both accept the existing diversity of formalisms and to provide means of organising their coexistence in a way that enables formal interoperability among ontologies.

**Figure 2:**
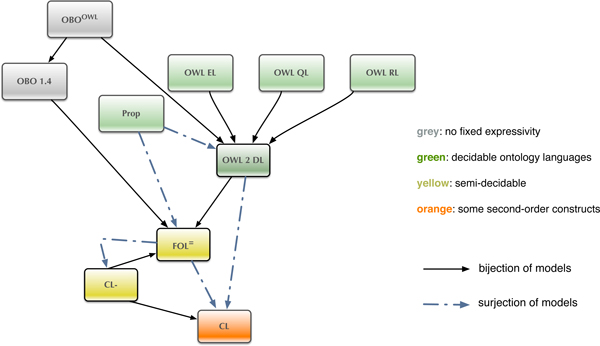
**The logic translation graph for basic ontology languages**.

At the heart of the DOL approach lies a graph of ontology languages and translations between them, displayed in Figure 2. (Compare [5] for the general theoretical background, and [67] for the proposed formal semantics. DOL is currently under standardisation as Working Draft ISO/WD 17347 in ISO/TC 37/SC 3 'Systems to manage terminology, knowledge and content'. See also http://ontolog.cim3.net/cgi-bin/wiki.pl?OntoIOp) This graph (here reduced to the languages discussed in this paper, the full graph and a discussion of the translations is presented in [66]) will enable users to

• relate ontologies that are written in different formalisms. E.g. prove that some OWL version of a biomedical ontology is logically entailed by its (reference) first-order version;

• re-use ontology modules even if they have been formulated in a different formalism;

• re-use ontology tools like theorem provers and module extractors along translations between formalisms.

Some of the DOL language features are as follows:

#### Usability

DOL allows for writing down ontologies and ontology links as implicitly as possible and as explicitly as needed; DOL allows for rich annotation and documentation of ontologies.

#### Syntax and expressivity

DOL has user- and machine-readable serializations and allows for expressing links between ontologies and for expressing heterogeneous ontologies.

#### Semantics

DOL has a well-defined formal, logic-based semantics; DOL is a logic-agnostic meta-language in the sense that its meta constructs are independent of the logical languages used in DOL modules expressed in various ontology languages.

#### Implementability/extensibility

DOL is intended to be generally applicable, open, and extensible; DOL should work with any existing or future ontology language (e.g. OWL, CL, F-logic, UML class diagrams, RDFS, and OBO).

DOL intends to cover all state-of-the-art basic ontology languages, and to provide a meta level on top of these. This meta level allows for the representation of logically heterogeneous ontologies in the sense that DOL ontologies may comprise of modules written in ontology languages with different underlying logics. Moreover, the DOL meta level constructs allow for logical links between ontologies such as relative interpretations or conservative extensions, mappings between ontologies (and their terms), merging operations, as well as non-monotonic extensions. The proposed meta-language intends to be an extensible framework for ontology languages, which means that any ontology language and any logic whose conformance with DOL has been established can be used with DOL. In particular, DOL establishes the conformance of a number of widely used ontology languages and logics as part of the standard; these include (ordered by increasing complexity) propositional logic (Prop), OWL [68] (with its profiles EL, RL and QL [69]), standard first-order logic with equality (FOL ^=^) and Common Logic (CL) [70], as well as translations between these ontology languages, displayed in Figure 2.

Note, in particular, that among these ontology languages, Common Logic is the most expressive one, and is therefore a target language for translations from all the other languages. Thus, when reasoning about heterogeneous distributed ontologies, one can prima facie translate all participating ontologies to Common Logic. However, note the difference to translating all ontologies to Common Logic in the first place: when, e.g., an OWL ontology has been translated to Common Logic, it is no longer easily amenable to decidable or even tractable reasoning procedures that OWL tools support. Therefore, DOL leaves all ontologies in their original formalisation to take advantage of the optimised automated reasoners for that particular language, and DOL tools should only translate them on demand. In summary, the functionality of the DOL framework may be captured by the following informal "equation":

$$\begin{array}{llll} \text{DOL} & =\text{meta}(\text{Prop},\text{EL},\text{QL},\text{OWL},\text{FO}{\text{L}}^{=},\text{CL},.\;.\;.\; & & \;\\ & \;\;\;\text{Prop}\to \text{OWL},\text{Prop}\to \text{FOL},\text{EL}\to \text{OWL},\text{OWL}\to \text{FOL},\text{FOL}\to \text{CL},.\;..)\; & & \;\\ & \; & \end{array}$$

where the meta(·) operation introduces the above mentioned meta-level constructs on top of the basic ontology languages using the built-in logic translations. A prototypical implementation of the DOL language is provided by the Hets system [71]. We next illustrate some of the DOL features by constructing a heterogeneous ontology for mereology.

### (Fragments of) mereology in biomedical ontologies

Certain phenotypic features can be described in terms of mereology. Indeed, as discussed in [72], part-whole relations are ubiquitous in biomedical ontologies. In fact, they typically employ both a *taxonomic *structure (is-a hierarchy) and a *partonomic *structure (part-of hierarchy).

Rather than repeating here the full syntax and semantics of the DOL language, we look in some detail at the example of specifying mereology for ontologies of different expressivity using different, heterogeneous formalisms, thereby illustrating some of the DOL features. While mereological relations such as parthood are frequently used in ontologies, many of these ontologies are formalised in languages that are not fully capable of *defining *the mereological notions. For example, mereological relations are often used in large biomedical ontologies which are implemented in the EL profile of OWL for efficiency. Similarly, Rector and Brandt [73] discuss what logical expressivity might be considered appropriate for the SNOMED ontology, and recommend to use the full OWL language to remedy various modelling problems found in SNOMED. However, even using the full expressivity of OWL is not sufficient for defining mereological relations, and more complete definitions require first-order or even second-order logic [5].

We now sketch a heterogeneous specification of mereology, introduced previously in different form in [5], using the DOL language.

#### Propositional

It starts with a Prop formalisation of the taxonomy of the categories over which DOLCE[39] defines mereological relations. We use syntax similar to OWL's Manchester syntax [74], which should be self-explanatory.

**distributed - ontology **Mereology

**logic **log : Propositional

**ontology **Taxonomy = *%% DOLCE' s basic taxonomic information about mereology*

**props **PT *%{ Particular} %*, PD *%{ Perdurant }%*, T *%{ TimeInterval }%*,

S *%{ SpaceRegion} %*, AR *%{ AbstractRegion }%*

. S ⋁ T ⋁ AR ⋁ PD → PT *%% PT is the top concept*

. S ⋀ T → ⊥ *%% PD, S , T, AR are pairwise disjoint*

. T ⋀ AR → ⊥ *%% remaining 'disjointness axioms' left out here . . *.

Although Prop is rarely regarded as an ontology language, this logic is quite popular for formal modelling since consistency and logical consequence can be quite efficiently decided using SAT solvers. In particular, for early detection of modelling errors in an ontology design (especially when using a large number of classes), initial consistency checks and satisfiability of classes can be delegated to a SAT solver for debugging.

#### *Description logic *EL *and interpretations*

We next specify a similar ontology in the EL profile of OWL (alternatively we could import the Prop ontology, see below), however adding a basic theory of 'parthood' by declaring it a as a transitive relation.

**logic **log : EL *%% syntax: OWL Manchester serialization/EL profile *[69]

**ontology **EL Parthood = *%% Parthood in OWL EL*

**Class **: Particular Category **SubClassOf**: PT *%% other class declarations omitted*

**DisjointUnionOf**: S, T, AR, PD *%% pairwise disjointness more compact*

**ObjectProperty **: is Part Of **Characteristics: Transitive**

As this ontology declares classes for all categories that are declared as propositional variables in the Prop ontology satisfying the same disjointness and subsumption relationships, the EL ontology *interprets *the Prop ontology (we here assume a Prop→OWL ontology language translation, compare [67]). Here, 'interpretation' is to be understood in the sense of model theory, i.e. the set of translated sentences of the source ontology have to logically follow from the target ontology. This is written in DOL and added to the distributed ontology as follows:

**interpretation **TaxonomyToELParthood : Taxonomy **with translation**

                                                               PropToOWL **to **ELParthood

**Figure 3:**
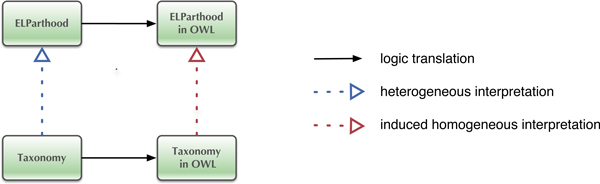
**Interpreting a taxonomy expressed in propositional logic in an EL ontology**.

Figure 3 illustrates the 'interpretation' construct in more detail. Note that we cannot in general directly translate propositional formulae to the OWL-EL fragment as EL, being a sub-Boolean logic, does not have full Boolean negation. Therefore, both the propositional theory and the EL theory will be implicitly translated to OWL, and the *induced homogeneous interpretation *will then verify whether or not the translated propositional formulae are logically entailed by the translated EL formulae.

#### Description logics: OWL-DL and beyond

We move on to add a module in the OWL-DL language. We import the EL parthood ontology and introduce additional parthood properties (such as asymmetry) which clearly go beyond the expressivity of Prop or EL.

**logic **log : OWL *                  %% syntax : OWL Manchester serialization *[74]

**ontology **ProperParthood = ELParthood **then**

**ObjectProperty **: is ProperPartOf **Characteristics **: **Asymmetric**

**SubPropertyOf **: isPartOf *%% Transitive + Asymmetric not allowed in OWL*

**Class **: Atom **EquivalentTo **: **inverse **isProperPartOf **only **owl : Nothing

                                    *%% an atom has no proper parts*

OWL still has quite good automatic reasoning support, but therefore also has its limits: OWL is not capable of defining e.g. the antisymmetry of the isPartOf relation. Overcoming such restrictions, we give 3 more extensions. Firstly in an expressive DL exceeding OWL expressivity, secondly in standard first-order logic FOL, and thirdly in Common Logic CL. First, the DL 'ExpDL' allows stronger definitions of several mereological notions by using the OWL language, but by violating some of OWL's syntactic restrictions, thus exceeding its expressivity (for a technical analysis see [75]). Note that the 'ExpDL' logic is not officially part of the DOL language, and here only presented for illustrative purposes; however, it can obviously be translated to FOL.

**logic **log : ExpDL *                  %% Parthood in DL exceeding OWL expressivity*

**ontology **Parthood = ProperParthood **then ***%% Parthood in OWL DL, as far as expressible*

**ObjectProperty**: is ProperPartOf **Characteristics : Transitive**

%% Transitive + Asymmetric not allowed in OWL

**SubPropertyOf **: isPartOf

**SubPropertyChain **: ispartof o isProperPartOf

                  *%% together with imported axioms violates OWL restrictions *[75]

Although already quite expressive, the logic 'ExpDL' still has no *explicit *existential quantification available, which makes it impossible to define notions such as overlap. This can however be easily done in first-order logic.

**logic **log : FOL *                  %% standard 'textbook' FOL*

**ontology **FOLParthood = Parthood **then**

∀ x,y : × . Ov (x,y) ↔ ∃ z : × . (isPartOf(z,x) ⋀ isPartOf (z,y))

                                    *%% definition of overlap*

#### Common logic CL

Finally, the CL ontology, which imports and extends the OWL and FOL ontologies (which are implicitly translated using the default FOL→CL and OWL→CL translations), gives a full specification of the mereology of 'classical extensional parthood' (see [39]). CL extends FOL with second-order style modelling, of which we use the possibility to quantify over predicates here. This allows us to concisely express the restriction of the variables *x*, *y*, and *z *to the same taxonomic category (perdurants, abstract regions, etc.), and it allows us to define a notion of second-order fusion [76].

**logic **log : CommonLogic            *%% syntax : CLIF dialect of Common Logic *[70, Annex A]

**ontology **ClassicalExtensionalParthood =

FOLParthood **then **{ *%% import OWL/FOL ontology from above , translate it to CL*

    . ( **for all **(X) (**if **(**or **(= × S) (= × T) (= × AR) (= × PD) )

         ( **for all **(x y z) (**if **(**and **(X x) (X y) (X z) )

                  ( **and **%% *now list all the axioms*

   ( **iff **( isAtomicPart Of × y) (**and **(isPartOf × y) (**Atom **x) ) )

   ( **iff **(sum z × y)

         ( **for all **(w) (**iff **(overlaps w z) (**and **(overlaps w x) (overlaps w y ) ) ) ) )

   ( **exists **( s ) ( sum s × y ) ) *%% existence of the sum *) ) ) ) )

. ( **for all **( Set a ) ( **iff **( fusion Set a ) *%% definition of fusion*

         ( **for all **(b) ( **iff **( overlaps b a )

            ( **exists **( c ) (**and **( Set c ) ( overlaps c a ) ) ) ) ) ) )

}

#### Reusing fragments of mereology in biomedical ontologies

**Figure 4:**
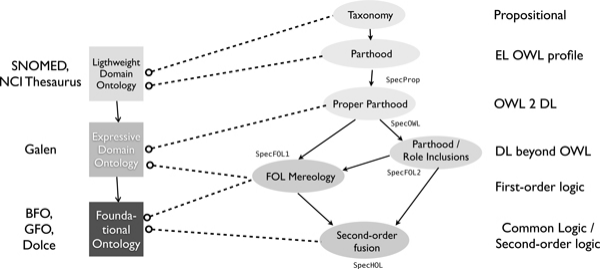
**Biomedical ontologies of different expressivity, importing different subtheories of the full mereology specification**.

Figure 4 serves two purposes: it illustrates graphically the structure of the heterogeneous specification sketched above, and it shows how the different modules can be re-used for ontologies of varying complexity and expressivity. Here, the lightweight EL ontology is extended to an OWL ontology that more fully axiomatises both Parthood and ProperParthood. This branches into two further extensions, namely a FOL ontology on the left, and an expressive DL ontology on the right that exceeds OWL expressivity (and which can be interpreted in the FOL ontology). Both may be extended to the CL specification. The lightweight EL axiomatisation might be imported (modulo renaming the classes over which we wish to specify e.g. parthood) in corresponding lightweight EL biomedical ontologies. For more expressive biomedical ontologies, both the OWL or the FOL specification may be important, depending on whether or not decidable reasoning is essential for the application of the ontology. FOL expressivity is of course often found in foundational ontologies, as are second-order features. However, even expressive theories of mereology are not sufficient for describing more complex issues in e.g. human anatomy on different levels of granularity, including holes or other topological notions, compare [77] for a discussion.

Note that apart from the constructs employed in the above mereology specification, namely the modular construction of ontologies using parts written in different formalisms (by importing along a logic translation) and the notion of interpretation, DOL comprises several other heterogeneous modelling features that are important for modelling purposes in biomedical ontologies, most notably *mapping *and *merging *of ontologies, as well as *non-monotonic extensions *which are discussed in more detail in the next section. The mapping facility allows to declaratively add the results of matching tools to an ontology specification, so called *alignments *(see [78] for an overview of matching research). Alignments are sets of correspondences, i.e. triples 〈*s, t, a*〉, where the term *s *from ontology *X *is considered *synonymous *with term *t *from ontology *Y*, adding a confidence value of *a *∈ [0,1]. In general, not only synonymy between terms may be considered as the 'linking relation', but also subsumption, parthood, or other relations. For instance, BioPortal (see http://bioportal.bioontology.org/) reports that the term 'Breast' from the NCI Thesaurus is mapped to the term 'mammary gland' from the Mouse adult gross anatomy ontology. For dealing with compositions of alignments in large biomedical ontologies, compare [35]. The Ontology Alignment Evaluation Initiative (OAEI) held at ESWC 2012 (see http://oaei.ontologymatching.org/) comprised two competitions evaluating the performance of ontology matching technologies for biomedical ontologies, one matching the Adult Mouse Anatomy (2744 classes) and the NCI The-saurus (3304 classes) describing the human anatomy, and one matching the Foundational Model of Anatomy (FMA) and the National Cancer Institute Thesaurus (NCI).

The **combine **operation in DOL, on the other hand, will use the specification of mappings identifying/differentiating terms from two distinct ontologies, and will return a new ontology that combines the axioms of both whilst respecting the identifications (see [79] for details as well as a discussion of the meaning shift that occurs when composing alignments). Such mappings of terms may, of course, be extracted from the results of corresponding matching tools, i.e. the obtained alignments. More use cases for the DOL language can be found in [80].

## HER2 phenotypes, mereology, and the non-monotonicity of normality

In this section, we discuss some aspects of the 'mereology of HER2', and the issue of 'normal' and 'abnormal' breast phenotypes. The *Human Epidermal growth factor Receptor 2 *(HER2) is an intramembrane protein that belongs to the family of epidermal growth factor receptors. HER2 positive breast cancer (HER2+ BC) has been recognised as a very aggressive form of cancer (see above), characterised by amplification and overexpression of the ErbB2 (HER2) gene as well as the genes of the ErbB2 amplicon. Accordingly, the HER2 protein, coded by the ErbB2 gene, is present in a high concentration within the cell. The related cell phenotype is characterised through various detection methods, e.g. immunohistochemistry (IHC), as showing a high concentration of the intramembrane receptor HER2, while the cancer tissue phenotype has been described as 'poorly differentiated' and associated with a poor prognosis. As the predictive power of HER2 has been demonstrated in clinical practice, HER2 has acquired additional descriptions such as tumour marker [81,82] and a drug target. 'Poor differentiation' of the examined tissue sample is still one of the main histopathological criteria for cancer assessment. However, as we focus here on the HER+ breast cancer, the feature that marks the disease is 'HER2 overexpression'.

### HER2 protein in normal and abnormal phenotypes

**Figure 5:**
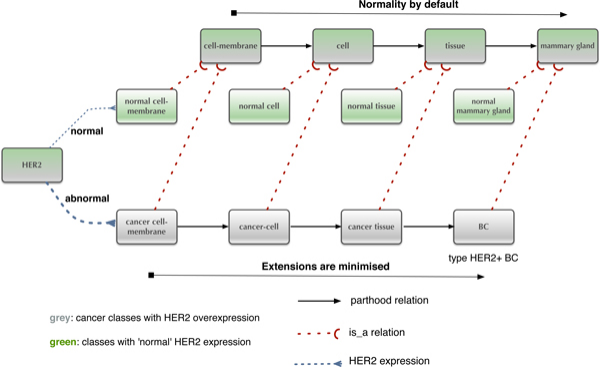
**HER2 as a difference maker: normal vs. abnormal breast phenotype**.

In our model (Figure 5), we describe a parthood relation in the context of a breast cancer subtype, where 'HER2' represents the intramembrane protein and a difference maker for the typical HER2+ breast cancer phenotypes. Each of the phenotype segments (i.e. the cell membrane, the cell, the tissue and the organ) acquires 'cancer phenotype' features due to overexpression of HER2.

Through a fine-grained representation of protein-protein interaction networks it can be captured how HER2 overexpression relates to carcinogenic processes, such as cancer cell growth, proliferation, migration, and adhesion that eventually result in a poorly differentiated tissue and an aggressive cancer phenotype. In our model, we abstract from the details of molecular processes, while using 'HER2' as a phenotype marker across the mereological segments.

**Figure 6:**
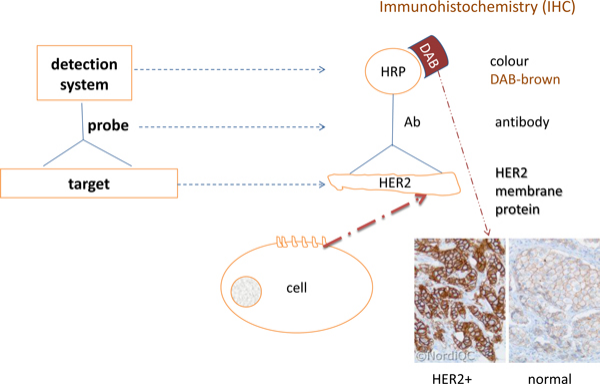
**HER2 protein detection by immunohistochemistry (IHC)**.

In terms of the Phene ontology [18], our model of HER2+ breast cancer includes *object-phene *structural classes of the cancer affected mammary gland (HER2+ breast cancer). Apparently, the relevant *quality *of the phenotype is *overexpression *of the HER2 protein. A detection system by a method such as immunohistochemistry (IHC) stains a tissue sample with a colour dye (see DAB in Figure 6). The produced image represents HER2 overexpression as 'brown colour' of the membrane.

Of course, the colour does not inhere in the HER2 protein. Rather, it marks the presence of the protein within the membrane. Thus, the *quality *of 'brown' membrane within the HER2+ breast tissue sample actually represents *quantity *of the HER2 proteins marked by the dye. As the quantity in this case describes the amount of proteins, we treat 'overexpression' in terms of detected HER2 proteins. The IHC method itself does not provide the number of HER2 proteins, but it just detects HER+cells in a tissue sample. However, it does not impact our model as quantitative proteomics and certain IHC image processing, in principle, can detect the amount of a protein (see e.g. [83,84]). For the same reason, our formal model, which considers IHC scores, assigns the 'cancer' feature to a phenotype on a tissue level. The detected number of HER2+ cell membranes corresponds to the number of HER2+ cells. A tissue sample having more than 30% of HER2+ cells acquires 'HER2+ cancer' phenotype [85], which is then also assigned to the mammary gland. Such an approach allows us to describe 'overexpression' as mereology of the HER2+ phenotype.

### Concept minimisation: normal vs. abnormal phenotypes

We have sketched in Figure 5 just a fragment of HER2 related breast cancer phenotypes. And indeed, note that the classification of organ parts etc. into *normal *vs. *abnormal*, as shown in Figure 5, is not a classical dichotomy as suggested by these terms. Indeed, attaching the property of abnormality to e.g. a particular tissue depends non-monotonically on the presence of information concerning overexpression of HER2. That is to say, unless such information is explicitly known, per default tissue will be considered normal. Therefore, adding information to the formal modelling (more instances of HER2 are part of some tissue) results in retracting the property of 'normality'.

Modelling this formally is not straightforward in standard ontology languages such as OWL or FOL. Or rather, it can only be simulated by explicitly listing exceptions. However, it can be done elegantly by using non-monotonic formalisms such as default rules [86], autoepistemic logic [87], or circumscription [88]. These formalisms were all devised in the early 80ies to overcome exactly such shortcomings of classical first-order logic in modelling exceptions and common sense rules.

Indeed, the necessity of supporting non-monotonic rules and abductive reasoning for ontology design has been noted many times (see e.g. [89-91]). In connection with biomedical ontologies, and phenotype ontologies in particular, Hoehndorf et al. [91] discuss the problem of combining an ontology for anatomy such as the Foundational Model of Anatomy (FMA) [92], which encodes a *canonical *view on its subject matter, with a phenotype ontology such as the Mammalian Phenotype Ontology [93], in which phenotype description often directly contradict the canonical definitions. A simple example is given by defining the anatomy of a mouse, which, canonically, always has as part a tail, whereas a 'mouse without a tail' is surely a reasonable description of a phenotype.

Unlike the approach of [91], using the DOL language allows to *declaratively *specify the non-monotonic extension of an ontology (or a combination of several ontologies) in one ontology specification. In the context of DOL, a multi-logic modelling environment, the non-monotonic framework of choice to be initially supported is *circumscription*, invented by John McCarthy in 1980. The reason for this choice, on a technical level, is simple enough to explain: the basic idea on which circumscription is based is the *minimisation *of (the extension of) concepts or relations (terms for short), assuming the extensions of some terms are fixed, whilst the extension of certain other terms may freely vary. To give formal semantics to this idea, all that is needed is the possibility to define a pre-order on models, which can be done in a logic-independent way.

Since we could here not sketch the semantics of non-monotonic extensions of DOL in sufficient detail, we only sketch how the HER2 modelling illustrated above in Figure 5 can be captured in a formal specification.

### Formal specification of a HER2 ontology

The HER2 ontology demonstrates how demands of the epistemic groups influence each other on a formal level. According to the St Gallen International Expert Consensus recommendation [85], the threshold for the determination of HER2 positivity by the IHC method is an intense membrane staining of >30% of the tumour cells. However, percentages can not directly be modelled in OWL (and for the purposes of this paper we refrain from using more expressive logics). Since percentage of staining is scored by pathologists and clinical oncologists, according to the panel of experts recommendations [94], into one of the classes 0, 1+, 2+, 3+, we here use a *qualitative *abstraction. I.e. we explicitly introduce the IHC HER2 Score as an enumerated concept.

The main idea now is to minimise the extension of the 'abnormal' concepts. I.e. unless there is evidence for overexpression of HER2 in, e.g., a tissue, it will be classified as 'normal'. The following specification sketches this idea.

**distributed**-**ontology **HER2

**logic **log :OWL

**ontology **HER2 = ProperParthood **then**

**Class **: IHC_HER2_Score

      **DisjointUnionOf **HER2_Negative , HER2_Borderline , HER2_Positive

**Class **: HER2_Negative **EquivalentTo **{0 , 1+}

**Class **: HER2_Borderline **EquivalentTo **{2+}

**Class **: HER2_Positive **EquivalentTo **{3+}

**Class **: HER2 **SubClassOf **Proteins

**Class **: Cell **DisjointUnionOf **: Normal_Cell , HER2+_Cell

**Class **: Tissue **DisjointUnionOf **: Normal_Tissue , HER2+_Tissue

**And **hasHER2Score **Exactly **1 IHC_Her2_Score

      *%% we assume all tissue to be IHC measured*

      *%% other disjointness axioms left out here *. . .

**Class **: Cell_Membrane **SubClassOf**

      isProperPartOf **Exactly **1 Cell

**Class **: HER2+_Tissue **EquivalentTo **Tissue **And **hasHER2Score **Some **HER2_Positive

      *%% determination of HER2 positivity of membrane by the IHC method*

**Class **: HER2+_Cell **SubClassOf **inverse isProperPartOf **Some **HER2+_Cell_Membrane

**Individual **: X

**Types **: Tissue **And **hasHER2Score **Some **{3+}

**Individual **: Y

**Types **: Tissue

**DifferentFrom **X

**minimise **HER2+_Cell_Membrane , HER2+_Cell , HER2+_Tissue

**vary **Normal_Cancer_Cell_Membrane , Normal_Cancer_Cell , Normal_Cancer-Tissue

      **then **%implies

**Individual **: X

**Types **: HER2+_Tissue

**Individual **: Y

**Types **: Normal_Tissue

**And **hasHER2Score **Some **( HER2_Negative **Or **HER2_Borderline )

            *%% Y is normal tissue (non-HER2 positive) without evidence to the contrary*

**Figure 7:**
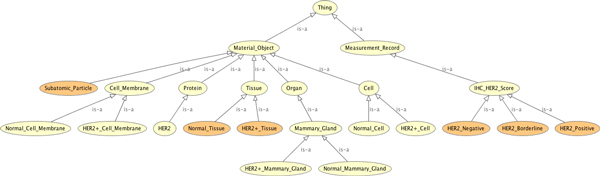
**The inferred class hierarchy of the HER2 ontology as displayed in Protégé**.

This ontology in particular assumes that any tissue is assigned an IHC HER2 Score, i.e. any particular tissue, if it were measured, would be assigned a specific value from IHC_HER2_Score. Here, the non-monotonicity comes into play. The minimisation of, e.g., the concept HER2+_Tissue means that any individual, here Y, which does not explicitly meet the defining criteria of HER2+_Tissue will be classified as belonging to its negation, i.e. being a Normal_Tissue. Without circumscription, no such new information could be derived (note that the statements following %implies are marked as following logically from the above ontology specification). Indeed, consider Figure 7 showing the class hierarchy of the HER2 ontology as implemented in OWL without circumscription. Whilst it does already infer that individual × must belong to the class HER2+_Tissue, it does not infer any information about individual Y.

Unrestricted use of circumscription easily yields undecidable reasoning when applied to even rather weak DLs. Whilst [89] discusses several such undecidability results and fragments of OWL for which it is decidable, there is also interesting restrictions that keep reasoning decidable even over full OWL [95].

### Implementation and future plans

We have discussed the importance of non-monotonic modelling for bio-medical ontologies, and have sketched a non-monotonic HER2 modelling, combining the theories of parthood and circumscription of concepts to minimise the 'abnormal' cancer-related concepts. The Hets system [71], which provides a prototypical implementation of DOL, is currently being extended to also support non-monotonic inference based on circumscription. The HER2 ontology is a work in progress, and we intend to include further criteria to describe 'abnormal' phenotypes, while reusing various OBO Foundry ontologies such as e.g. the Units of measurement ontology (see http://obofoundry.org/cgi-bin/detail.cgi?id=unit), and the Phene ontology [18] classes and relations. In particular, we aim at performing an experimental verification of the usefulness of the model. Existing ontology repositories such as BioPortal lack the ability to host heterogeneous ontologies such as the HER2 ontology just sketched. We therefore host the HER2 ontology at OntoHub (see http://ontohub.org/), a new ontology repository currently under active development that will support DOL ontologies and their features in their full generality. Users of OntoHub can upload, browse, search and annotate basic ontologies in various languages via a web frontend. OntoHub accesses the Heterogeneous Tool Set Hets via a RESTful web service interface for having the structure of ontologies analyzed. Hets already supports a large number of basic ontology languages and logics, and is capable of describing the structural outline of an ontology from the perspective of DOL, which is not committed to one particular logic (see [80] for more information on OntoHub). Beyond basic ontologies, OntoHub supports linking ontologies across ontology languages, and creating distributed ontologies as sets of basic ontologies and links among them. An important difference to the mapping facilities of e.g. BioPortal is that links in OntoHub have formal semantics, and therefore enable new reasoning and interoperability scenarios between ontologies.

## Concluding remarks

We tackled the problem of phenotype representation from various perspectives. As our main interest is in merging knowledge from different domains, we addressed some of the 'domain knowledge problems' of [96] through inspecting a number of examples from molecular oncology and clinical practice. In particular, we demonstrated that a breast cancer phenotype ontology needs to deal with a plurality of biomedical representations as well as onto-logical formalisms.

In order to clarify how domain specific representations can be integrated on a formal level, we distinguished six epistemic groups involved in the construction of a knowledge base. The criteria used for the classification of the different groups were the representational means and the degree of representational and semantic explicitness. The introduced distinctions illustrate how an intertwined collaborative effort leads experts from diverse domains to establish a formalised and explicit knowledge representation. We stressed the need for a more detailed characterisation of domain specific epistemic interests, including a deeper understanding of the characterised groups (1)-(6), in order to provide a more sustainable integration of knowledge about breast cancer, increasing interoperability of represented information and, therefore, applicability of acquired clinical and biological knowledge. A closer understanding of the domain needs would also further support decisions about which formalisms best suit a domain.

Thus, instead of favouring a unifying approach to knowledge integration, our analysis rather shows that heterogeneous representations, which originate from different epistemic needs, also ask for a representational pluralism. A pluralism of perspectives on a problem, as exemplified in breast cancer phenotype ontology, can integrate fruitful insights from various domains, and e.g. enable the application of molecular knowledge into clinical practice.

Concerning formal representations, dealing with the heterogeneities of phenotypes, we propose to endorse a framework that allows to organise the various (domain) representations into an interlinked modular structure, respecting the plurality of formalisms, expressivities and aims, as they are found across diverse scientific communities. In particular, we have described a modelling of defeasible inference based on circumscription that allows to model the normal/abnormal distinction in phenotypes in a transparent way.

We have proposed the DOL language to address some of the interoperability needs in ontology design, and, indeed, we believe that no attempt at an integration of knowledge can be epistemically sustainable unless it embraces the plurality of formal languages and tools, methodologies and perspectives, as they result from the heterogeneity of domain interests. DOL is intended to support just such a pluralistic integration of domain interests on a formal level, namely *Open Biomedical Pluralism*.

## Competing interests

The authors declare that they have no competing interests.

## Authors' contributions

The distinction between different epistemic groups and the discussion of perspectivism in phenotype representations and phenotype ontologies is due to AS. The presentation of the distributed ontology language DOL is due to OK. Other parts including the HER2 ontology have been developed jointly. All authors read and approved the final manuscript.
